# *Capnocytophaga canimorsus* tricuspid valve endocarditis

**DOI:** 10.1016/j.idcr.2021.e01083

**Published:** 2021-03-24

**Authors:** Sienna Lindén, Patrik Gilje, Johan Tham, Sandra Lindstedt, Magnus Rasmussen

**Affiliations:** a Department of Clinical Sciences Lund, Division of Infection Medicine, Lund University, Lund, Sweden; b Department of Clinical Sciences Lund, Division of Cardiology, Lund University, Lund, Sweden; c Skåne University Hospital, Lund, Sweden; d Department of Translational Medicine, Division of Clinical Infection Medicine, Lund University, Malmö, Sweden; e Skåne University Hospital, Malmö, Sweden; f Deptartment of of Clinical Sciences Lund, Division of Cardiothoracic Surgery and Transplantation, Lund University, Lund, Sweden; g Wallenberg Center for Molecular Medicine, Lund University, Sweden; h Lund Stem Cell Center, Lund University, Sweden

**Keywords:** *Capnocytophaga canimorsus*, Infective endocarditis, Tricuspid valve

## Abstract

*Capnocytophaga canimorsus* is an uncommon cause of infective endocarditis (IE) and mainly affects persons with compromised immune-systems who have been in contact with dogs. We describe a case of *C. canimorsus* tricuspid valve IE in a 70 year-old dog-owner where diagnosis and treatment were delayed. The reason for the delayed diagnosis in this case was likely due to that initial blood cultures were negative due to preceding antibiotic treatment, discrepancies between echocardiographic investigations, and a thymoma and colonic polyps which were thought to explain the symptoms. A multi-diciplinary approach in cases with suspected IE might help to avoid diagnostic delays.

## Introduction

*Capnocytophaga canimorsus*, is a bacterium present in the oral flora of healthy dogs and cats [1]. It is a slow growing, fastidious gram-negative rod. Previous difficulties in species determination have been overcome with the introduction of MALDI TOF-MS in microbiological laboratories [2]. In humans, *C. canimorsus* is an opportunistic pathogen most commonly infecting individuals with predisposing conditions. It has been described to cause sepsis, especially in asplenic patients or alcoholics, but it can also cause meningitis, septic arthritis and rarer so, infective endocarditis (IE) [3,4]. The diagnosis of IE depends on culturing the bacteria from blood and on the detection of endocardial changes upon echocardiography [5]. *C. carnimorsus* is a rare cause of IE and knowledge of this infection comes from case reports only. A detailed literature review is accounted for after the presentation of our case.

## Case report

A 70-year-old woman, with a history of osteoarthritis in both hands, visited the emergency room (ER) with a 3-day history of fever. Two weeks prior to this she had developed rashes on her abdomen and mouth but had no other focal symptoms. A systolic murmur was noticed and the patient described that this had been already noted during her childhood. Workup was unremarkable and a viral infection was suspected.

The patient returned to the ER five days later due to continued fever. A temperature of 38.4 °C was noted, CRP was 123 mg/L and small excoriations were noted around the mouth. A clinical diagnosis of impetigo was made. The cultures taken from the mouth sores demonstrated growth of *Staphylococcus aureus* and blood cultures were negative. The patient was treated with flucloxacillin, 1 g three times daily for ten days.

**Fig. 1:**
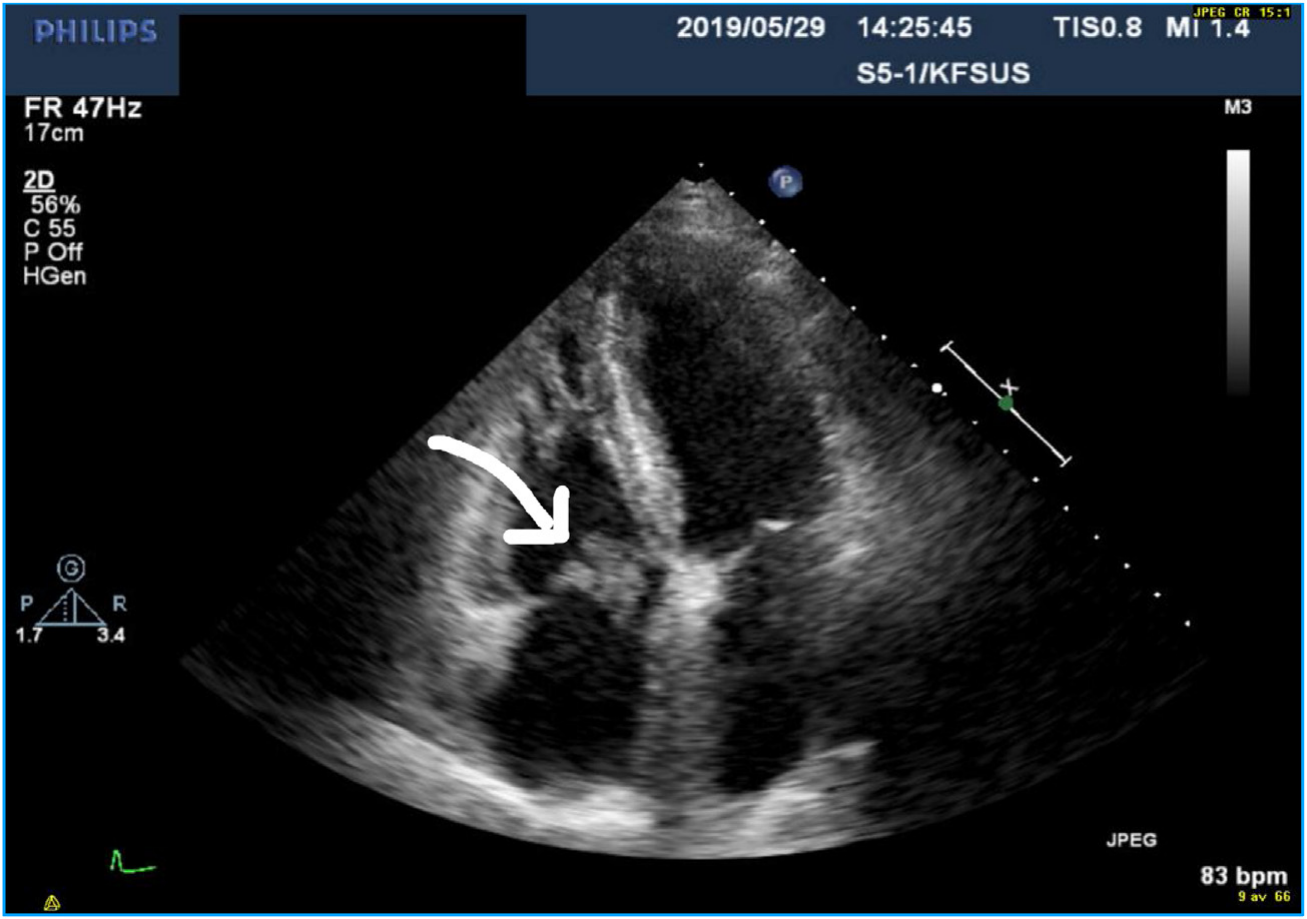
Transthoracic echocardiography demonstrating tricuspid vegetation (arrow).

**Fig. 2:**
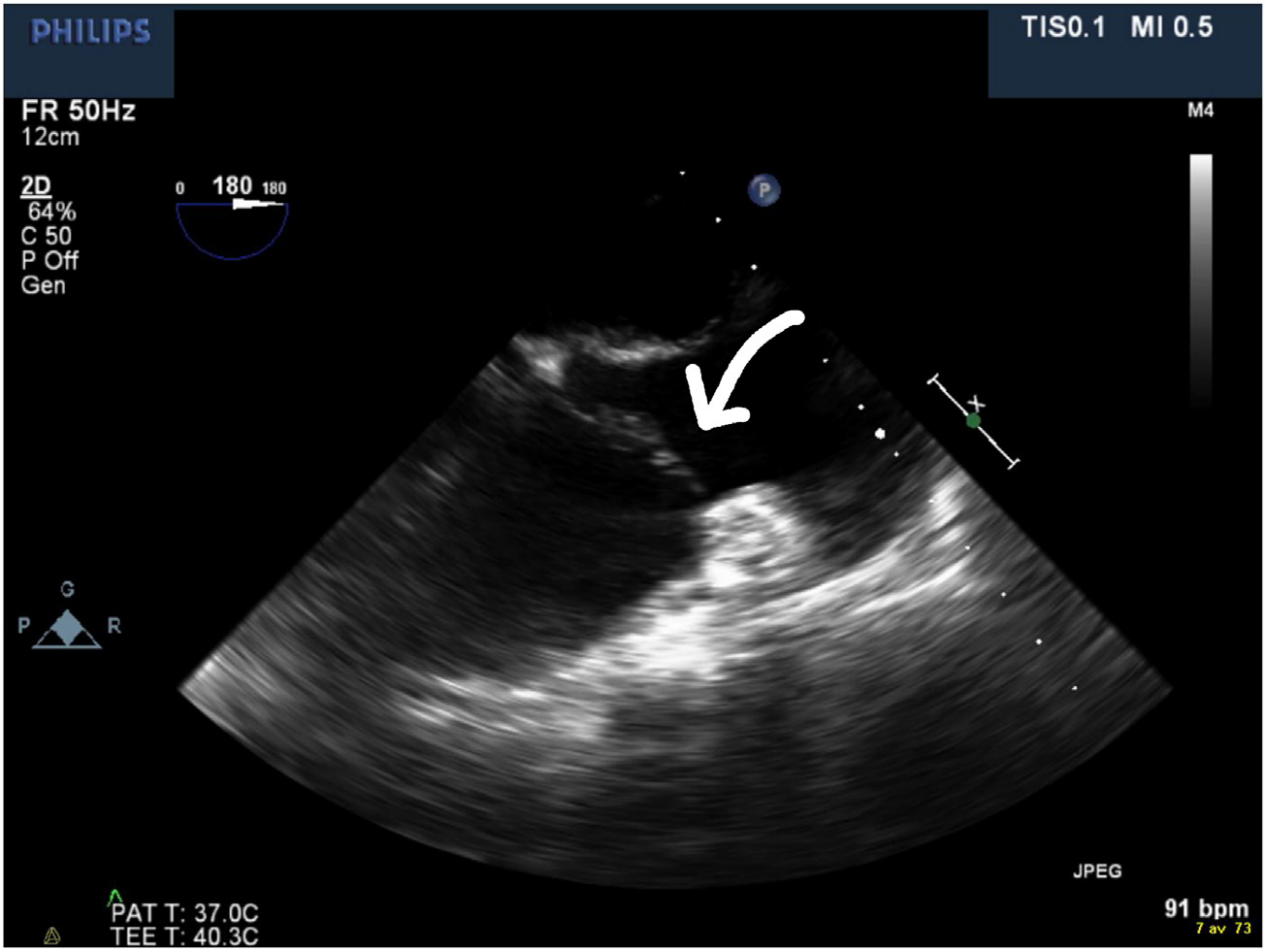
Transesophageal echocardiography five days after Fig. 1. No vegetation can be seen.

**Fig. 3:**
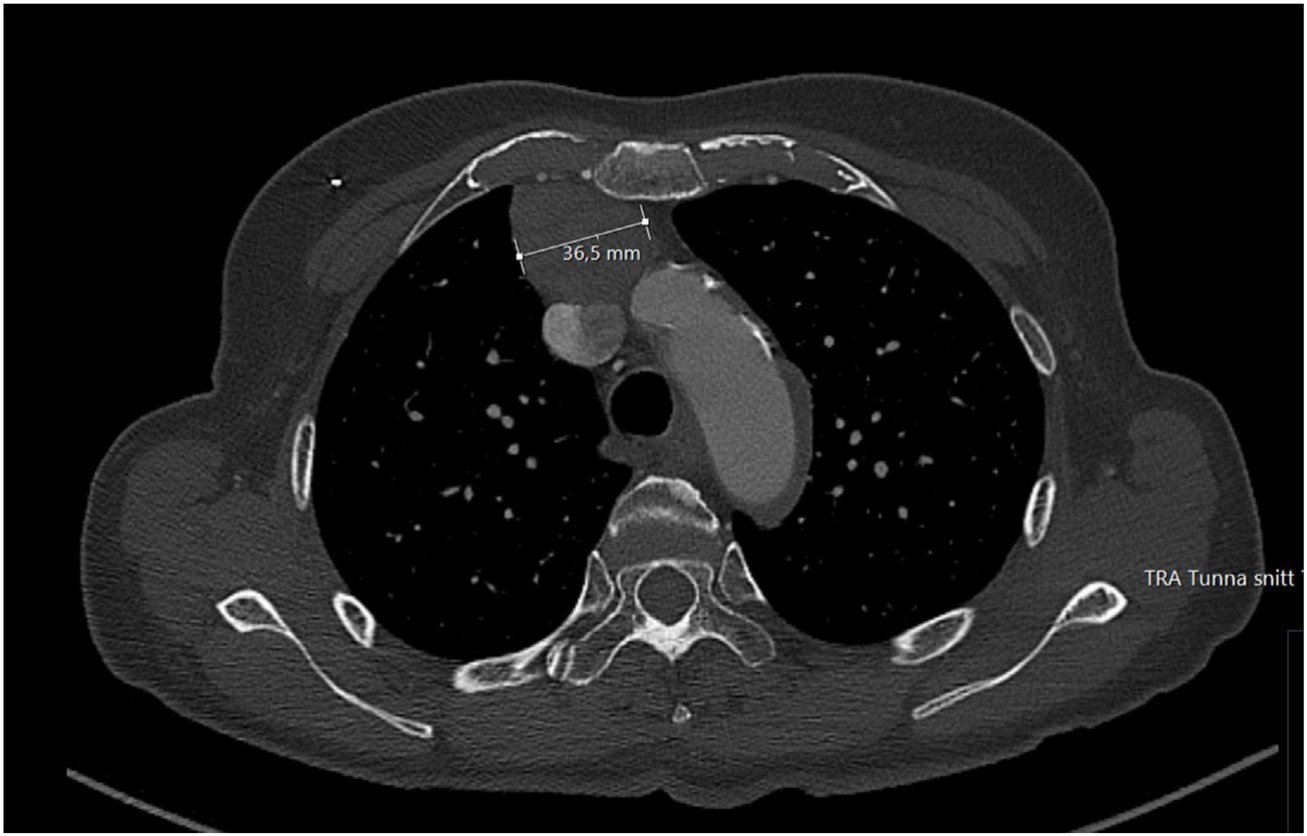
Computed tomography demonstrating mediastinal tumour.

Eleven days later, the patient returned to the ER again and was admitted due to the persistent fever. Blood cultures were drawn while the patient was still on flucloxacillin. Leukocyte count was 16,000 cells/μL with 15,000 neutrophils/μL, CRP was 85 mg/L and ESR was 58 mm. An echocardiogram was performed first transthoracically (TTE) and five days later transesophageally (TEE). TTE revealed a mobile structure (12 × 14 mm) highly suggestive of a vegetation on the tricuspid valve (Fig. 1). Five days later a TEE was performed which showed an unspecific thickening of the tricuspid valve and mild tricuspid regurgitation but no typical vegetation (Fig. 2). Since blood cultures were negative and TEE inconclusive, additional work-up was performed. A pulmonary X-ray demonstrated an enlarged mediastinum, and thus a computed tomography (CT) of thorax was performed revealing bilateral pulmonary embolism, enlarged mediastinal lymph nodes and a soft tissue tumor of 4.5 × 4 × 3 cm in the mediastinum (Fig. 3). Treatment with 100 mg enoxaparin once daily subcutaneously was initiated. No antimicrobials were given. During the hospital stay, CRP rose from 85 to 177 mg/L but the patient was clinically stable and wished to leave the hospital for further out-patient follow-up. Repeated blood cultures were negative. Further diagnostic workup was performed at a specialized policlinic at the oncology department.

An FDG PET-CT was performed and showed a moderate uptake of the mediastinal tumor as well as in the lungs corresponding to the location of the pulmonary emboli previously noted on CT scan. A focal hypermetabolism was also seen in the caecum which was suspected to be an adenoma. To obtain a diagnosis of the mediastinal tumour a middle-sized-needle biopsy guided by a CT scan was performed and revealed a histological and immunohistochemical picture compatible with a thymoma. An endobronchial ultrasound with transbronchial needle aspiration from the enlarged lymph nodes was performed and cytology was benign.

The condition of the patient worsened with continuous fever, malaise, weight loss and fatigue. A colonoscopy was performed two months after initial presentation and a 2 cm lobulated polyp from the ascending colon and one broad based polyp from the sigmoid colon were removed. Histology report displayed low-grade dysplasia and no signs of malignancy. At colonoscopy the patient was found to be in a poor general condition with dyspnea at rest and pitting edema of the legs. The lungs were auscultated with bilateral rattles.

The patient was again admitted but was afebrile. Haemoglobin was 8.1 g/dL, CRP was 167 mg/L and white cell blood count was 19,000 cells/μl. Blood cultures were obtained and all bottles showed growth of *C. canimorsus*. Species determination was performed using MALDI-TOF MS with a score of 2.1. ETESTs were performed using Müller-Hinton fastidious agar (MHF) and demonstrated a minimal inhibitory concentration of 0.032 μg/mL for benzylpenicillin. Thus, intravenous benzylpenicillin treatment (5000 units (three grams) four times daily) was initiated. The patient was asked about animal contact and acknowledged that she had dogs that had licked her skin. However, she assured that she had not been bitten.

**Fig. 4:**
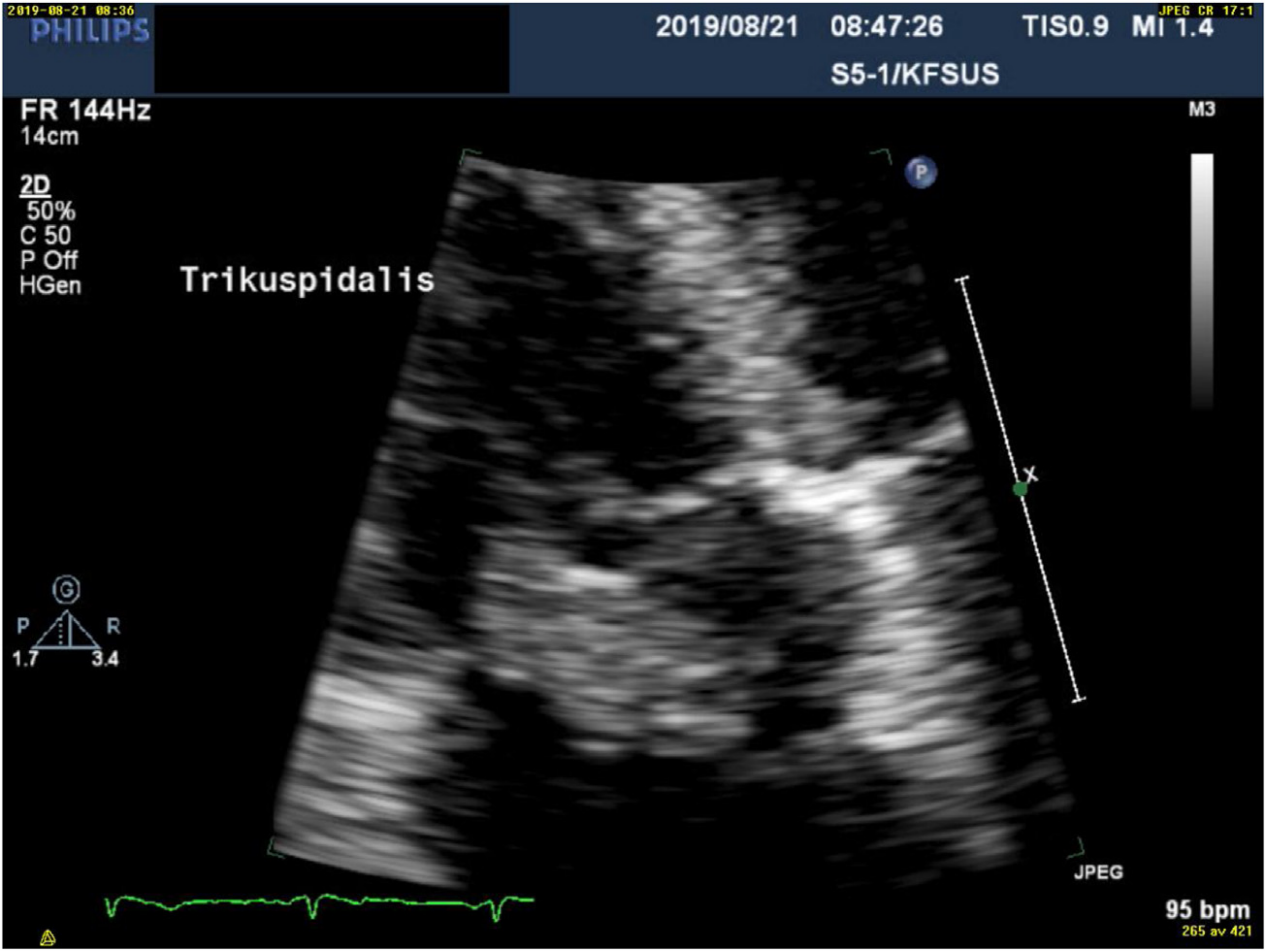
Transhoracic echocardiography demonstrating enlarged tricuspid vegetation.

Repeat TEE demonstrated a large and mobile vegetation (20 × 30 mm) on a partially destroyed tricuspid valve with severe tricuspid regurgitation (Fig. 4). A multi-diciplinary endocarditis conference deemed surgery necessary and the patient successfully underwent biological tricuspid valve replacement and concomitantly a transsternal thymectomy. Postoperatively the patient showed signs of right heart failure and the TTE revealed moderately reduced right ventricular function, necessitating two days of diuretics and inotropic drugs. Cultures from the excised valve showed no growth, but DNA from *C. canimorsus* was detected using 16S rRNA gene PCR followed by sequencing. The histological examination of the thymoma demonstrated that the resection was radical. Post-operative antimicrobial treatment consisted of two weeks of benzylpenicillin. Postoperative recovery was slow but uneventful. A TTE three weeks after surgery demonstrated mildly reduced right ventricle function and a normal biological tricuspid valve. The patient felt fully recovered, and there were no signs of inflammation at a check-up four months after surgery.

## Discussion

*C. canimorsus* is an uncommon cause of IE and reported cases are summarized in Table 1. A common theme of described cases is immune-suppression and dog contact. Our patient had dogs and it appears possible that the thymoma might have rendered her at least slightly immune supressed.

**Table 1:** Summary of the clinical features of patients with IE caused by *C. canimorsus*.

Sex	Age	Previous conditions	Location of IE	Echo findings	Animal Contact	Antibiotic	Surgery	Outcome	Refs.
M^a^	73	PAV, AF	PAV	VEG	Dog	Mer	No	Sup	[14]
M	50	–	AV	NK	Dog Bite	NK	Yes	Died	[1]
NK	NK	NK	AV	NK	NK	PcG	Yes	Rec	[1]
NK	NK	NK	MV	NK	NK	PcG	No	Rec	[1]
M	64	NK	TV, AV	VEG	Dog Bite	Van + Gen	No	Died	[6]
M	43	ALC	MV, AV	VEG	Lion Bite	NK	MVR	Died	[15]
M	42	ALC	AV	ARA	Dog Bite	NK	MVR	Rec	[16]
M	52	AS	AV	VEG	Dog Bite	PcG + Gen	No	Rec	[17]
M	56	–	TV	VEG	Dog	PcG + Gen	No	Rec	[8]
M	47	ALC	TV	VEG	Dog	Van + Gen	TV repair	Rec	[7]
M	63	PAV	PAV	ARA, Re	Snogged dog	PcG	BVR, PM	Rec	[18]
M	65	AS	AV	ARA, VEG	NK	Cef, PcG + Gen	BVRR	Rec	[19]
M	55	COPD, ALC, IVDU	AV, TV	ARA, fistula, VEG	Dog	Mer + Cip	TV repair, MVR	Rec	[9]
F	69	COPD	TV	VEG, Re	None	Var	No	Rec	[10]
M	39	ALC	MV	VEG	Dog	Amp + Tob	No	Rec	[20]
F	41	MS	MV	Re	Dog Bite	Cef	MVR	Rec	[21]
M	46	–	AV	VEG, Re	Dog Bite	Cef	BVR	Rec	[22]
F	76	ICD	ICD	VEG	Dog scratch	–	ICD extraction	Rec	[23]
F	47	NK	TV	VEG	Dog faeces	Mer	BVR	Rec	[11]
M	56	None	NK	NK^17^	Dog	PcG + Gen	No	Rec	[24]

a M, male; NK, not known; PAV, prosthetic aortic valve; AF, atrial fibrillation; Mer, meropenem; Sup, chronic suppression; AV, aortic valve; Rec, recovered; MV, mitral valve; TV, tricuspid valve; Van, vancomycin; Gen, gentamicin; PcG, penicillin G; ALC, alcoholism; AS, aortic stenosis; COPD, chronic obstructive pulmonary disease; IVDU, intravenous drug use; MS, mitral valve stenosis; VEG, vegetation; ARA, aortic root abscess; Re, regurgitation; Cip, ciprofloxacin; Amp, Ampicillin; Tob, tobramycin; MVR, mechanical valve replacement; BVR, biological valve replacement; PM, pacemaker implantation; Var, various; BVRR biological valve and root replacement.

At least six previous case reports have described tricuspid valve IE with *C. canimorsus* and thus this uncommon IE location constitutes a large share of the described cases [[6], [7], [8], [9]]. For other types of IE pathogens, causing subacute infection, this IE location is very rare [12]. Tricuspid valve IE is otherwise an infection affecting intravenous drug users and is typically caused by *S. aureus* [13].

The initial clinical presentation of our patient with persisting fever, tricuspid valve vegetation, clear signs of inflammation and pulmonary embolism was of course highly indicative of IE although criteria for definite IE were not fulfilled [5]. Three factors possibly contributed to the erroneous decision not to treat the patient for IE. The first factor was that blood cultures were negative and even repeated cultures after one week without antimicrobials were negative. The fact that blood cultures can be negative during and after antimicrobial therapy is well recognized, especially for alpha-hemolytic streptococci. In this case, despite that the patient received treatment with flucloxacillin which likely has poor activity against *C. canimorsus*, blood cultures remained negative for more than a week without treatment. In hindsight, it would have been correct to treat the patient as a case of blood culture negative possible IE at this point. The second factor that contributed to the delay of diagnosis was that the vegetation noted on TTE could not be clearly visualized on TEE five days later. The reason for this was likely that the vegetation seen on TTE embolized to the lungs and therefore was not left on the valves when TEE was performed. The lung emboli were later visualized by the CT scan and were likely caused by the embolized vegetation. The third factor that contributed to the failure to diagnose and treat the patient for IE was the finding of the thymoma. For the specialized oncology policlinic, the task became to plan the work-up and treatment of a potentially malignant tumour. However, the sudden onset of fever together with signs of inflammation could hardly be explained by the thymoma. The FDG PET-CT contributed even more to the confusion when an uptake in the colon was noted.

When the patient finally was correctly diagnosed she had developed symptoms of heart failure, the valve was destroyed with severe insufficiency, and the vegetation was very large. Therefore, surgery was deemed necessary and the finding of DNA from *C. canimorsus* in valve tissue confirmed the diagnosis of IE.

This case demonstrates that despite being a cornerstone in the diagnosis of IE, blood cultures are not always positive in this condition. This is especially true when the patient has received antimicrobials to which the bacteria are sensitive. Furthermore, the possibility of embolization should be taken into consideration when a TTE with high suspicion of endocarditis is followed up by a negative TEE. Finally, careful analysis of patient history, signs and symptoms are often more important to achieve a correct diagnosis than technically advanced investigations.

## Funding

No specific funding was available for this work.

## Author contribution

**Sienna Lindén:** Case presentation, literature review, drafting of manuscript. **Patrik Gilje**: Data analysis and echocardiography expertise. **Johan Tham**: input on clinical features and on microbiology. **Sandra Lindstedt**: input on surgical treatment. **Magnus Rasmussen**: Case presentation, literature review, correspondence.

All authors gave input to case presentation and approved to the final version of the manuscript.

## Ethical approval

Not applicable according to Swedish law.

## Informed consent

The patient gave informed consent to the publication of this report.

## Declaration of Competing Interest

The authors report no declarations of interest.
